# Randomized controlled trial for the efficacy of three versus five sessions of grief counseling on the psychological aspects following COVID-19 bereavement: A study protocol

**DOI:** 10.3389/fpsyt.2022.1047448

**Published:** 2022-12-05

**Authors:** Ahmad Hajebi, Maryam Rasoulian, Marjan Fathi, Amir Tiyuri, Maryam Abbasinejad, Morteza Naserbakht, Ali Asadi, Nooshin Khademoreza

**Affiliations:** ^1^Research Center for Addiction & Risky Behaviors (ReCARB), Psychosocial Health Research Institute, Iran University of Medical Sciences, Tehran, Iran; ^2^Department of Psychiatry, School of Medicine, Iran University of Medical Sciences, Tehran, Iran; ^3^Mental Health Research Center, Psychosocial Health Research Institute, Iran University of Medical Sciences, Tehran, Iran; ^4^Health Deputy, Iran University of Medical Sciences, Tehran, Iran; ^5^Department of Epidemiology, School of Public Health, Iran University of Medical Sciences, Tehran, Iran; ^6^Department for Mental Health and Substance Abuse, Ministry of Health and Medical Education, Tehran, Iran; ^7^Mental Health Research Center, Psychosocial Health Research Institute, School of Behavioral Sciences and Mental Health, Iran University of Medical Sciences, Tehran, Iran

**Keywords:** grief, counseling, COVID-19, bereavement, mental health

## Abstract

**Background:**

During the COVID-19 pandemic, many people have experienced traumatic losses and therefore are at risk of developing complicated grief regarding the restrictions on the performance of routine mourning rituals. This study is a randomized controlled trial for assessing the efficacy of three versus five sessions of grief counseling on grief intensity, psychological distress, and quality of life of grief among bereaved people due to COVID-19.

**Methods:**

A total of 120 bereaved people, due to COVID-19, will be enrolled in this multi-center randomized controlled trial after assessment for inclusion and exclusion criteria. Following the informed consent procedure, participants will be allocated into two groups equally by the Stratified Balanced Block Randomization, one of them delivering a three-session grief counseling intervention and the other delivering a five-session grief counseling intervention. The intervention will be delivered by trained psychologists *via* in-person individual sessions. The primary outcome is grief intensity, and the secondary outcomes are psychological distress, quality of life, and satisfaction of the participants. These outcomes will be measured by the Grief Intensity Scale (GIS), the General Health Questionnaire-28 (GHQ-28), the Short Form Health Survey-12 (SF-12), and the Client Satisfaction Questionnaire (CSQ-8), respectively. The assessments will be done at three time points, one before the intervention and the others 1 month and 3 months after the intervention. The data will be analyzed using the SPSS V.18 and Stata V.11 software. The analysis approach will be “intention to treat.”

**Discussion:**

Results of this study can be applied for selecting the most suitable intervention leading to the prevention of complicated grief and the maintenance and promotion of the mental health of bereaved people due to COVID-19.

**Clinical trial registration:**

[irct.ir], identifier [IRCT20200505047305N1].

## Introduction

Grief is an emotional reaction manifesting as deep sadness and regret and a range of other feelings which an individual experiences following the loss of a loved one (1). The concept of grief is mainly related to the various reactions and mental feelings that individuals experience after a loss and especially death of an intimate person (2, 3). Grieving individuals may not ever return to their previous emotional status but are usually able to go on with their lives and let go of their pain and eventually start to build new relationships (4, 5). This process happens in a process named bereavement. Bereavement is a universal reaction to loss, and individuals come to an acceptance of it over a period of about 6 months to 1 year; their attachment to the lost one loosens as they come back to normal life (6, 7). Although grief is a normal reaction, it is accompanied by various feelings. Some are able to handle the loss and cope with it through the mourning process, and others experience severe sorrow throughout a long period. These severe emotional reactions are poor prognostic factors and predictors of a prolonged grief disorder or complicated grief (8). Based on International Classification of Diseases 11th Revision (ICD-11), prolonged grief disorder is a disturbance in which, following the death of a person close to the bereaved, there is persistent and pervasive grief response (more than 6 months at a minimum) and causes significant impairment in personal, family, social, educational, occupational, or other important areas of functioning (9). The DSM-5 (diagnostic and statistical manual of mental disorders, fifth edition) proposed “persistent complex bereavement disorder” as a psychiatric disorder categorized under the entity of stress-related disorders (1). Individuals diagnosed with persistent complex bereavement disorder are also at an increased risk of mental health and other health problems. This may also affect their behavior and at times lead to suicidal ideation or attempts, necessitating therapeutic interventions (10). We know that COVID-19 has many physical and mental consequences, including lung, kidney and liver complications, drug-related complications, depression, anxiety, and psychological distress (11–14). The high prevalence of psychological distress, stress reaction, insomnia symptoms, and the increased burden of mental disorders during the pandemic provide evidence for the serious impact of COVID-19 on mental health (15, 16).

Ever since the COVID-19 outbreak began, many people have been experiencing loss and are going through tragic processes of shock and denial (17, 18). Evidence shows that following sudden and unpredictable deaths such as deaths due to COVID-19, grief levels are higher, and grief reactions are more intense and, therefore, may convert to complex or prolonged grief disorder (8, 19–22). In this case, Tang and Xiang reported a high prevalence of prolonged grief disorder among people bereaved due to COVID-19 (23). These bereaved people have to face fears of virus transmission during corpse handling, which leads to limitations in the burial process alongside their deep anguish, and they have no opportunities to say goodbye (21, 24). Social distancing measures simultaneously hinder the performance of routine mourning rituals and ceremonies, which otherwise would have a facilitating role in the grieving process for the individual. Mourning is a societal process, and bereaving individuals are in need of social support to be able to go through it; nevertheless, in COVID-19 cases, social support is mainly absent (24–26).

Grief counseling is a psychological intervention that assists bereaving individuals manage their emotions throughout the grief process (8). Evidence-based interventions for reducing the burden of suffering on bereaved individuals have been proven to decrease long-term health risks and are beneficial in improving clinically relevant outcomes (27). Psychological interventions in grieving individuals have both immediate and long-term effects on grief, especially when delivered individually (28). Greater symptom change has been observed in people at risk for developing persistent complex bereavement disorder in the long term and after the intervention has been completed, and this shows that counseling helps more than the passage of time alone (29). For individuals diagnosed with persistent complex bereavement disorder, psychological interventions are quite different and are consisted of specific techniques (8).

The study aims at comparing the efficacy of providing three versus five sessions of a grief counseling intervention to family members of those deceased due to COVID-19 in the setting of a multi-center randomized clinical trial. The primary outcome measure is the intensity of grief of individuals after a 3-month follow-up period. Quality of life, psychological distress, and satisfaction with the intervention are the secondary outcomes that will be compared among both groups. In this study, in addition to a general investigation of the efficacy of grief counseling, we aimed to compare the efficacy of three- and five-session grief counseling. If similar efficacy is seen between the two groups due to a lack of resources, three-session counseling can be introduced to the authorities as a population-based intervention for bereaved people in the country. In this case, by spending less time and money, more bereaved people will benefit from this service.

We hypothesize that the effect of the three-session grief counseling intervention on the grief intensity, psychological distress, quality of life, and satisfaction with the intervention among participants will be comparable with the five-session intervention and supposedly have equal efficacy.

## Methods

### Study design

This study is a parallel multi-center randomized controlled trial for comparing the efficacy of a three- versus five-session counseling intervention on bereaved people who had lost a loved one due to COVID-19 by dividing them into two equal intervention groups.

### Inclusion and exclusion criteria

The participants are first-degree relatives of those deceased due to COVID-19, ranging from 15 to 64 years old. According to the study protocol, following 1 week after the occurrence of the death of a COVID-19 patient, a phone call will be made to connect with a family member of the deceased to initially assess them regarding the inclusion criteria. If the individual is eligible and willing to participate, the grief counseling sessions will be started 2 weeks after the death of the loved one. Participants should be literate and be able to understand and speak the Persian language. They should all give written informed consent to enter the study. If an individual has received any type of mental health service, including biological or non-biological therapies or interventions throughout this 2-week period, or if the individual has a severe comorbid medical or neurological condition along with disability, or fulfills the criteria of any psychiatric disorder, they will be excluded from the study. Serious suicidal ideation, suicidal attempts and a history of self-harm, and being diagnosed with any substance use disorder are also among the exclusion criteria. If a participant enters the counseling process and any of these exclusion criteria come up in the middle of the study; in that case, that individual will be excluded from the study but the necessary and standard therapy will not be withheld.

### Setting

This study will be conducted in the setting of 20 health centers affiliated with 10 of the universities of medical sciences of Iran, which are responsible for health services and medical education in their catchment area (including Ahvaz, Arak, Golestan, Guilan, Iran, Kerman, Mashhad, Shiraz, Tabriz, and Tehran universities). In each university, two health centers will be selected. These centers routinely offer basic mental health services by master clinical psychologists. A specific number of clinical psychologists of these centers will be further trained in a 2-day online workshop. In this workshop, psychologists learned how to perform three- and five-session mourning counseling according to a protocol by an expert panel to provide the intervention in a uniform and coordinated manner.

### Intervention

The content of grief counseling was provided by an expert panel, including psychiatrists and psychologists with experience in this field. Its stages include a literature review about the method of mourning during the COVID-19 pandemic, psychosocial consequences of COVID-19, types of grief, interventions related to grief counseling and treatment of traumatic bereavement, preparation of an initial intervention package with an emphasis on the COVID-19 pandemic, editing the package after the implementation of the pilot intervention and based on the opinions of the interveners and the target group. A service package has been developed to help the bereaved people of those deceased due to COVID-19 to go through the normal grieving process. This intervention package mainly focuses on the items below:

- To facilitate the acknowledgment and acceptance of the recent loss.
- To help the participant cope with the pain and anguish of the recent loss.
- To assess the participants’ defense mechanisms and approaches toward the recent loss.
- To mutually explore finding some sort of meaning in the painful event.
- To empower the participants to manage difficult situations and adjust to normal life.
- To enable participants to be able to live in the absence of the lost one and to learn coping and problem-solving skills.
- To find a way to be comfortable in life while keeping bonds with the lost one.

In both intervention methods, each session takes 45 min. Each session starts and ends with reviewing and presenting assignments. In both intervention groups, if the participant misses a session, the psychologist will call them two times at two different times on two different days to ask about the reason for their absence in the session. In case the participant is not willing to cooperate further, they will be excluded from the study.

#### The five-session intervention group

In the five-session intervention, sessions will be held once a week, and the topics of the sessions will be as follows:

1. Session 1: Psychological assessment and preliminary consultation; creating a therapeutic alliance, and collecting demographic information.
2. Session 2: Open evaluation of the event and acceptance of the loss; narrative of death (talking about the events related to death by the bereaved), mourning narrative (talking about the mourning ceremony), and evaluating the dominant emotions such as anger, disappointment, anxiety, sadness, and missing.
3. Session 3: Learning to cope with the loss and searching for meaning in loss; explanation about the usual methods of dealing with grief: effective and inefficient methods, discussing the bereaved confrontation with the grief, and explanations about the meaning.
4. Session 4: Recovery and adjustment to normal life in the absence of the deceased; the role of the deceased person in the life of the bereaved, the problems ahead after the loss, reviewing the usual method of solving problems, and teaching problem-solving techniques.
5. Session 5: Assessment of the bereaving process and returning to life in the absence of the lost one; examining the feelings and emotions experienced, feelings about self, examining behavioral changes in self-care and relationships, and examining the change in attitude toward goal setting for the future.

#### The three-session intervention group

In the three-session intervention, sessions will be held every 2 weeks, and the topics of the sessions will also be as follows:

1. Session 1: Psychological assessment and preliminary consultation; creating a therapeutic alliance and collecting demographic information.
2. Session 2: Open evaluation of the event and acceptance of the loss, expanding understanding of the recent loss, learning to cope with the loss and searching for meaning in loss; flexible assessment of the event and increased understanding of the reality of loss/coping with grief, increasing understanding of the reality of loss and coping with the pain caused by loss, examining defenses and countermeasures, and helping survivors find meaning in painful experiences.
3. Session 3: Recovery and adjustment to normal life in the absence of the lost one; recovery, adaptation, and return to life without the deceased, helping survivors adjust to loss through problem-solving, returning to life, and examining the changes according to their narrative.

### Recruitment process

Five of the researchers will be in direct connection with the 10 medical universities. In every university, two health centers will be selected for service delivery, and one trained clinical psychologist will be the service provider in each center. The clinical psychologist obtains names and phone numbers of bereaved people on a routine basis from the social service department of the hospitals in the catchment area of the center and will invite them by phone to encourage them to attend the grief counseling sessions. The clinical psychologist will also try to create effective communication and express compassion and empathy toward the grieving individuals. When participants attend health centers, their demographic data are obtained and registered, and the inclusion or exclusion criteria will then be re-assessed. If inclusion criteria exist and in the absence of the exclusion criteria, the participant will be thoroughly informed on the designed counseling intervention process, and informed consent will be obtained. If several family members of one COVID-19 victim attend the health center to receive service, only one of them will be enrolled in the study, and the rest will be directed to receive the usual grief counseling services provided at health centers. Assessments will be done at three time points, one before the intervention and the others 1 month and 3 months after the intervention.

### Consent procedure

Participants entering the study will receive oral explanations of the trial intervention and will sign an informed consent form. This is performed by the clinical psychologist of the health center, and they are responsible for responding to any queries. Informed consent will be mainly provided in a general non-technical language, so that it would be comprehensible at any scholastic level. If a participant does not have the necessary inclusion criteria or does not give informed consent to be enrolled in the study, they will be excluded from the study and will receive services as usual. The informed consent method of this study has been approved by the Medical Ethics Committee of the Iran University of Medical Sciences (IUMS) and has been registered with the trial registration number of IRCT20200505047305N1. Participants can exit the trial whenever they wish.

### Randomization

After informed consent, participants are randomized into three- or five-session intervention groups. Allocation of participants to each group will be done with the use of Stratified Balanced Block Randomization. Twelve participants will be selected from each university, and randomization will be done separately for each. Among 20 possibilities for six blocks including forms of three individuals in the five-session group and three individuals in the three-session group, two blocks will be randomly chosen for each university in the Microsoft Office Excel software with the use of the RANDBETWEEN formula and a random sequence will be formed. Subsequently, participants will be divided into the three- and five-session groups equally according to the random sequence. Randomization will be done in the main research headquarters in the Mental Health Research Center of the IUMS, and information will be only given to the health centers at the time of initiation of the intervention.

### Data collection/management process

The primary outcome measure is the grief intensity based on the grief intensity scale (GIS) score of individuals receiving grief counseling sessions after a 3-month follow-up period, and the secondary outcomes are the quality of life, psychological distress, and satisfaction with the services. In this study, one qualified clinical psychologist will be entitled to each medical university attending two of the health centers to collect data at three points of participant entry (baseline assessment) and two follow-up points at 1-month and 3-month follow-up periods. Satisfaction with the services will be assessed at the end of the counseling sessions (either three or five) and 3-month after it. Clinical psychologists who are working as raters will be trained to use the questionnaires, and inter-rater reliability will also be checked after the training sessions. To reduce the risk of research bias, the raters will not be among those clinical psychologists providing the grief counseling sessions and will be blind toward the number of sessions each individual has received.

Demographic variables will be obtained using a questionnaire created by the researcher, and data collection for obtaining the dependent variables of the study will be performed by using the GIS, the General Health Questionnaire (GHQ-28), the Short Form Health Survey (SF-12), and the Client Satisfaction Questionnaire (CSQ-8).

#### The grief intensity scale

The GIS is a scale proposed by Prigerson et al. (30) and consists of 12 questions measuring the thoughts, emotions, and behaviors of individuals who have recently lost an important person. This scale represents the severity of the reactions of the bereaving individual. It facilitates clinicians to be able to assess the risk of an individual being later diagnosed with prolonged grief disorder after the loss of a loved one. The first two questions of this scale question the time elapsed since the death of a loved one and the decline in performance. The sum of the scores of the next 10 Likert-scale questions, each of which is answered with never, at least once a month, once a week, once a day, and several times a day, is analyzed as a grief intensity score. Each item receives a score of 1–5. A higher score represents a higher intensity of grief symptoms (31). We conducted a pilot study to investigate the validity and reliability of the Persian version of GIS and estimate the mean and standard deviation of the grief intensity score of the bereaved persons due to COVID-19. The face and content validity of the questionnaire was confirmed by the expert panel. Cronbach’s α of 0.92 and the intraclass correlation coefficient of 0.87 indicated good internal consistency and test–retest reliability of the Persian version of GIS.

#### The general health questionnaire

The GHQ-28 is designed for screening non-psychotic mental disorders and is commonly used among researchers all over the world. It consists of four subscales, such as somatic symptoms, anxiety symptoms, social functioning, and depressive symptoms. The Farsi version of the GHQ-28 has been validated by Noorbala et al. (32) for use in Iranians above 15 years old, and it has demonstrated good reliability and validity for research. The 28-item questionnaire includes multiple-choice questions accompanied by the following four possible responses: Not at all, No more than usual, Rather more than usual, and Much more than usual. A score ranging from 0 to 3 can be given for each response, with a total possible score ranging from 0 to 84. A higher total score is an indicator of poor general health, and 23 is the most reliable cut-off point for the presence of distress (32).

#### The short form health survey

The SF-12 is a short form of the SF-36 Health Survey with 12 questions and two main domains that provide glimpses into mental and physical functioning, including physical functioning (two items), bodily pain (two items), limitations in usual role activities because of physical problems (one item), general health (one item), vitality and energy (one item), social functioning (one item), limitations in usual role activities because of emotional problems (two items), and perceived mental health (two items). The individual’s score is calculated separately for the mental and physical component summary. The SF-12 has been validated for use in the Iranian population by Montazeri et al. (33), demonstrating Cronbach’s α of 0.72 and good reliability and validity.

#### The client satisfaction questionnaire

The CSQ-8 is a questionnaire proposed by Larsen et al., which is used to measure clients’ satisfaction rate with health services (34). This questionnaire consists of eight items, each question is accompanied by four responses from very positive to very negative, and each item gets a score of 1–4. The minimum total score is 8, and the maximum total score is 32. A higher score shows higher satisfaction with the services. The internal consistency of the questionnaire has been validated with Cronbach’s α of 0.91 (35). In the study of Imanzadeh et al. (36), the validity and reliability of the Persian version of CSQ-8 were approved.

#### Reducing loss to follow-up

For decreasing loss to follow-up of the participants, their phone numbers will be obtained at the beginning of the counseling sessions, and before each assessment, they will be reminded by phone 1 week before. If the participant does not attend, two follow-up telephone calls will be made. Calls will be at different times on different days, so the possibility of reaching the participant will increase. If, after two telephone calls, the participant is not willing to cooperate, the rater will try to fill out the GIS by phone and end the assessment.

### Blinding

It is obvious that we will not be able to blind the participants of the two groups toward the number of sessions, but the raters will not be among those clinical psychologists providing the grief counseling sessions and will be blind toward the number of sessions each individual has received. Those who are involved in the analysis of the data will also be blinded toward the participants’ affiliation to each group, as these data will be coded.

### Quality assurance

Before the study, the service providers will be trained in a 2-day workshop to be able to offer the trial service package. Raters will also be trained to fill out the questionnaires. One team will train the whole team of raters and service providers, and one single training module will be used. Quality assurance at the national level will be the responsibility of the “National Executive Committee.” One supervisor will be chosen from each university which will ensure keeping up with the study protocols and standards, including calls, counseling sessions, and ratings. One national coordinator will also be allocated for every two universities for further supervision.

### Sample size calculation

The sample size has been calculated to be 60 individuals in each group, which is a total of 120 individuals. Twelve individuals will be selected from two health centers of each of the 10 universities. This calculation has been done using the G*Power software (37) for comparing the grief intensity score as the primary outcome among the two groups and based on the results of the pilot study. In this calculation, the type one error (α) is 0.05, the type two error (β) is 0.2, the effect size (d) is 0.5, and the drop-out expectation is 20% of the participants.

### Planned analysis

The data, after entering and cleaning, will be analyzed using the SPSS V.18 and Stata V.11 software. Mean and standard deviation will be calculated to continuous data and frequency, and percent will be used for showing categorical variables. The analysis approach will be “intention to treat.” An independent and paired *t*-test, a chi-square, and a repeated-measures ANOVA (non-parametric tests if necessary) will be used to compare the studied outcomes in the follow-ups between and within the groups. The generalized estimating equations (GEEs) will be fitted in separate models for each outcome, and α < 0.05 will be considered as statistical significance.

### Ethical considerations

Before the study, the participants will be fully informed about the study and research process, and informed consent will be obtained from every individual. Confidentiality will be reassured, and the results will not contain personal identification data. Participants will be ensured that they can drop out of the study at any point of the study and receive the routine services. Participants will not have to pay for the services. The questionnaires will be labeled with a code, and data will be entered into the database without names. The informed consent process has been approved by the Ethics Committee of IUMS with the code of IR.IUMS.REC.1399.272 and has been registered with the trial registration number of IRCT20200505047305N1.

## Discussion

This study is a multi-center randomized controlled trial for comparing the efficacy of a three-session versus a five-session counseling intervention on the bereaved people of those deceased due to COVID-19 regarding the intensity of grief, psychological distress, quality of life, and satisfaction with the services. In the recent COVID-19 outbreak, many people lost their loved ones without being able to perform mourning ceremonies due to safety protocols and were deprived of the necessary social support they could have received otherwise from their family members and friends, putting them at risk of later developing complicated and prolonged grief disorder as health crisis due to COVID-19 (21, 23, 38). This study is a multi-center randomized controlled trial with the objective of preventing psychological problems in bereaved people due to COVID-19, being conducted for the first time in Iran. In addition to a general investigation of the efficacy of grief counseling, we aimed to compare the efficacy of three- and five-session grief counseling. If similar efficacy is seen between the two interventions, the three-session counseling can be introduced to the authorities as a population-based intervention for bereaved people in the country. In this case, by spending less time and money, more bereaved people will benefit from this service.

The results of this study will facilitate policymakers, planners, clinicians, and other service providers to design proper interventions for the prevention of complicated and unexpressed grief among the bereaved people due to COVID-19, and this can have a vast effect on the mental health of the society in the COVID-19 and post-COVID-19 era.

## Ethics statement

The studies involving human participants were reviewed and approved by the Ethics Committee of Iran University of Medical Sciences (Reference number IR.IUMS.REC.1399.272). The patients/participants provided their written informed consent to participate in this study.

## Author contributions

AH and NK designed the study and procured the funding. MR, MF, MN, MA, and AA monitored the study at the various study sites. AT and MN analyzed and interpreted the data and wrote up the reports. NK had the ultimate authority over all of these activities. AH, MR, NK, and AT wrote the draft of the manuscript. All authors collaborated in developing the plan for the trial, contributed to the article, and approved the final version of this manuscript.
